# Pessimistic dairy calves are more vulnerable to pain-induced anhedonia

**DOI:** 10.1371/journal.pone.0242100

**Published:** 2020-11-18

**Authors:** Benjamin Lecorps, Emeline Nogues, Marina A. G. von Keyserlingk, Daniel M. Weary

**Affiliations:** Animal Welfare Program, Faculty of Land and Food Systems, University of British Columbia, Vancouver, British Columbia, Canada; Michigan State University, UNITED STATES

## Abstract

Pain induces deficits in appreciation of rewards (i.e. anhedonia) and variation in response to pain may be partly explained by individual differences in general expectations (i.e. optimism). Dairy calves are routinely subjected to painful procedures such as hot-iron disbudding. We tested if female Holstein calves (n = 17) display signs of anhedonia (as evidenced by reduced consumption of a sweet solution) after hot-iron disbudding (performed under general and local anesthesia), and whether individual differences in optimism explain the variation in this response. Individual variation in optimism was measured using responses to two judgment bias tests (performed when calves were 25 d old), and anhedonia was measured by comparing consumption of a sweet solution before and after hot-iron disbudding. We found that intake of the sweet solution declined (by mean ± SD: 48.4 ± 44.3%) on the day after disbudding, and that more pessimistic calves were more affected. Sweet solution consumption did not return to baseline for the duration of the study (i.e. 5 days). Calves reduced their intake of a sweet solution after hot-iron disbudding, consistent with pain-induced anhedonia, and more pessimistic calves showed stronger evidence of anhedonia, suggesting that they were more affected by the procedure. However, our results cannot rule out the possibility that calf responses were driven by anorexia.

## Introduction

Pain is defined as “a distressing experience associated with actual or potential tissue damage with sensory, emotional, cognitive, and social components” [1] and, in non-human animals, is often assessed using basic behavioral (e.g. wound-directed behaviors) and physiological responses (e.g. changes in glucocorticoid levels) [2]. These measures can be useful to assess the intensity and location of the pain, but do not allow strong inferences regarding the affective component. Pain has effects on cognition in humans, including information processing and decision-making [3]. Cognitive changes include cognitive biases, defined as alterations in the perception and interpretation of situations [4], and anhedonia, defined as “*deficits in the hedonic response to rewards*” [5]. Anhedonia is one of the most studied behavioral changes associated with depression in humans and may also provide insight into pain-induced affective experiences in animals.

Given its subjective nature, the experience of pain varies from one individual to another [6]. Pain is not only a matter of afferent inputs, but rather a complex and integrated response [7, 8], so differences in pain sensitivity may originate from any stage of pain processing, including psychological and cognitive processes [6]. For instance in humans, higher levels of optimism are associated with increased pain tolerance [7, 9] and susceptibility to placebo [10], indicating a link between general expectations and pain perception. No studies to date have explored this relationship in animals. In fact, individual differences in response to pain have been largely ignored in non-human animals, even though a better understanding of variation in pain responses may improve the validity of animal models [11] and the ability to mitigate the negative effects of painful procedures.

Hot-iron disbudding is routinely performed on dairy calves, and despite recent efforts to promote the use of pain control (e.g. Canadian dairy code of practice), the procedure is mostly performed with limited or no pain control [12, 13]. Thus, the procedure provides the opportunity for researchers to study pain without imposing new harms. Hot-iron disbudding leads to the expression of wound-directed behaviors and to increased cortisol levels, responses that can be mitigated using intra-operative (i.e. general and local anesthesia) and post-operative (e.g. non-steroidal anti-inflammatory drugs) pain control [14]. Previous studies showed that hot-iron disbudding is aversive [15] and induces negative mood (i.e. calves became more pessimistic towards ambiguous cues [16]). Some recent evidence suggests that the latter response may be due to a lowered motivation/pleasure associated with accessing the milk reward (i.e. an anhedonia-like response; [17]) but, no study to date specifically aimed to explore whether hot-iron disbudding affects the perception of hedonic experiences.

The consumption of a sweet solution is commonly used to assess anhedonia in laboratory rodents [18]. Evidence suggests that sucrose is rewarding for cattle [19], especially in calves [20], a phenomenon that seems well-conserved across species (invertebrates [21], pigs [22], horses [23] and rodents [24]). In this study we used changes in the consumption of a sweet solution (5% sucrose) to infer pain-induced anhedonia after hot-iron disbudding. We expected that calves would decrease their consumption of the sweet solution following hot-iron disbudding, indicating pain-induced anhedonia.

Interest in animal personality is increasing, mostly because it provides an understanding of why individuals vary in response to a similar situation [25, 26]. Stable inter-individual differences in optimism have recently been described in non-human animals [27, 28], including dairy calves [29]. Optimism seems to modulate responses to stressors in both humans [30] and non-human animals [27, 31]. Considering that no work to date has focused on the individual response to disbudding, we tested whether more pessimistic calves are more vulnerable to pain (i.e. as evidenced by greater pain-induced anhedonia).

## Materials and methods

The study was approved by The University of British Columbia’s Animal Care Committee (#A16-0310-A002). All animals were housed and disbudded as part of standard farm and industry practices.

### Animals and housing

Twenty healthy Holstein female calves (mean ± SD birth weight: 39.0 ± 4.6 kg) were housed in two groups of 10 animals (mean age range: 16.5 d) in 25 m^2^ pens bedded with sawdust where they had access to 12 L/d of pasteurized whole milk via an automated milk feeder (one teat; CF 1000 CS Combi; DeLaval Inc., Sweden), and *ad libitum* access to water, hay and grain.

### Experimental procedures

**Fig 1:**
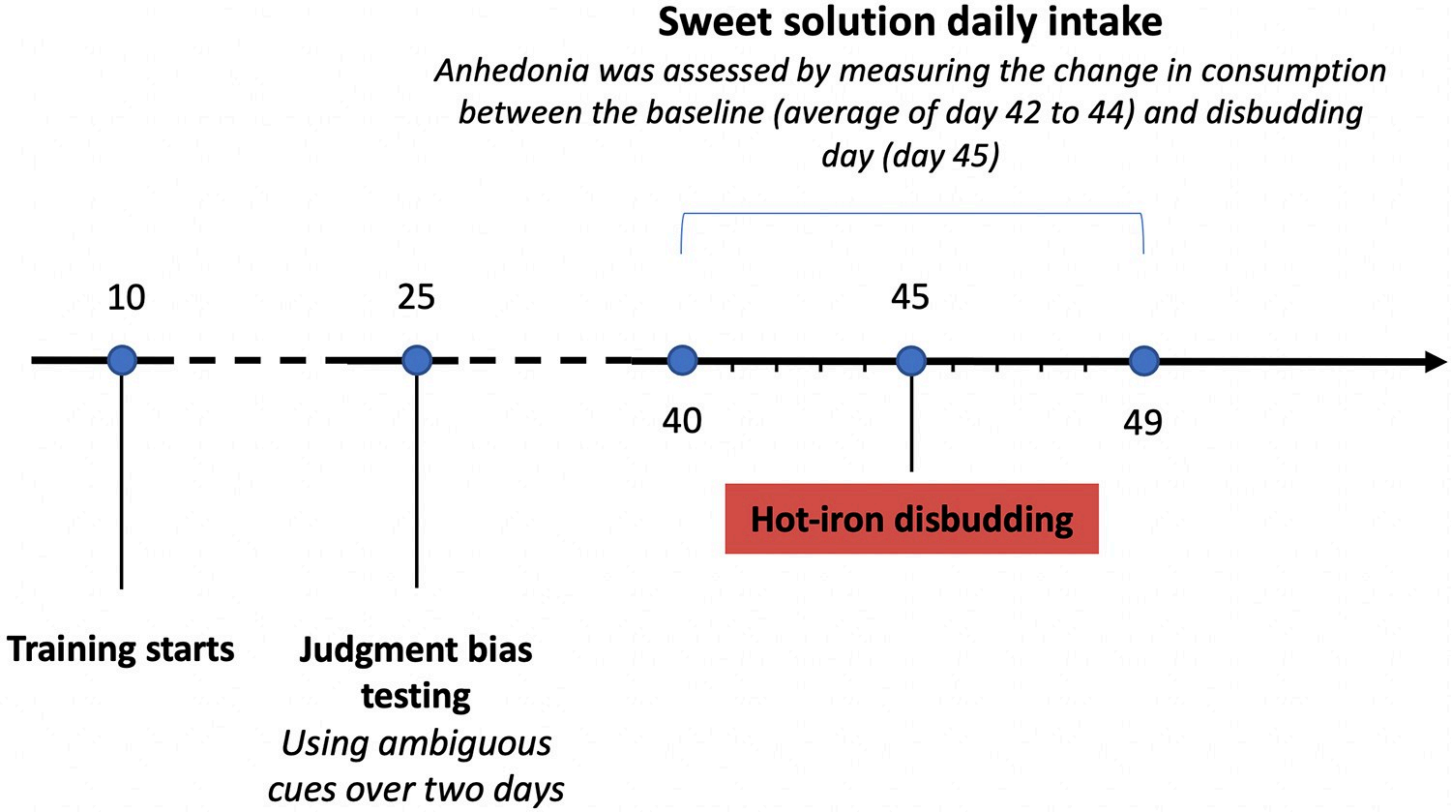
Timeline of the experimental procedure. Calves (n = 20) were trained for the judgment bias tests and then tested over 2 days starting at 25 d of age. The latency to touch each of the 3 ambiguous locations was averaged. Calves were offered *ad libitum* access to a sweet solution for 6 h/d (from 16:00 to 22:00) in their home-pen and daily intakes were measured from 40 d to 49 d of age. Disbudding occurred on d 45 at 10:00.

Calves were tested at specific ages for judgment bias and anhedonia (see Fig 1).

### Training and testing for judgment bias

Calves were individually brought from their home pen and placed in an experimental arena composed of a start box connected to a 16m^2^ sawdust bedded area, similar to that described by Lecorps et al. [29]. During training, calves learned to associate one side of the apparatus with a reward (i.e. milk) and the other side with a mild punishment (empty bottle + air puff). Calves were first trained (starting at 10 d old) to associate one side with the reward over 5 trials on each of 3 consecutive days. The second step of training consisted of pseudo-random presentations of bottles on the rewarded and punished sides. Training required approximately 12 ± 2 d until calves met the learning criterion (2 consecutive days without errors). Once trained, calves were tested using three ambiguous locations (i.e. bottles placed between the rewarded and punished locations); these locations were labelled “Near positive (nS+)”, “Middle (M)” and “Near negative (nS-)”, and were positioned 0.75 m, 1.5 m and 2.25 m away from the rewarded location, respectively. Testing was carried over two days when calves were approx. 25 days old; each location was presented once per day in a pseudo-random order, always starting with one of the reinforced locations (i.e. rewarded or punished). In each trial, calves were allowed up to 30 s to touch the bottles and the latency to touch was recorded. If a calf failed to approach within 30 s it was returned to the start box and 30 s was recorded as the approach latency. Ambiguous locations were never rewarded or punished. We chose to limit calves’ exposure to ambiguous cues to prevent habituation [32]; a previous study using the same design over 4 days did not find evidence of habituation [29]. To ensure calves were motivated to participate to the task, access to milk ended at 22:00 on days before training and testing sessions.

### Anhedonia

Starting at least 10 d before hot-iron disbudding, calves were given *ad libitum* access to an unflavored sweet solution (concentration: 50 g/L sucrose providing 200 kcal/L; this concentration was found to be effective in pilot work preceding this study) for 6 h/d (between 16:00 and 22:00) in their home-pen using a second automatic feeder (RIC, Insentec B. V., Marknesse, the Netherlands), allowing one calf to drink at a time. Calves had no previous experience with this feeder. Calf’s identity and intake (in kg) were automatically recorded at each visit. Daily intakes were collected 5 d before and after hot-iron disbudding (see Fig 1). To encourage calves to drink the sweet solution, milk allowance was reduced by 25% when calves were 40 d old based on the volume consumed during the 3 preceding days.

### Hot-iron disbudding

Calves were disbudded in their home-pen at 45 ± 0.7 d old at 10:00 h. Calves were provided 0.2 mg/kg of xylazine (SC, right rump, Rompun 20 mg/mL, Bayer, Leverkusen, Germany) as a sedative, followed by a cornual nerve block on each horn (5mL per side of 2% Lidocaine; Ayerst Veterinary Labs, Ontario) as a local anesthetic. 10 min later, calves were tested for pain responses with a needle-prick (none responded) and then disbudded using a hot-iron (X30, 1.3 cm tip, Rhinehart, Spencerville, IN, USA) positioned over the horn bud for multiple short periods (total contact time of approximately 15 s). The calf was then positioned in sternal recumbency and allowed to recover.

### Statistical analysis

A previous study showed that calves were consistent in their response to judgment bias tests using a sample size of 22 animals [29]. Here, we used 20 animals considering that a sample size of 15 individuals was recommended for power set at 0.8, significance level set at 0.05 and a Cohen’s d equal to 0.8. Calves were considered the statistical unit. Model residuals were scrutinized for outliers and normality. In cases where the normality assumption was not met, transformations were applied as described below.

Responses to ambiguous cues typically follow a generalization gradient [32]. Thus, calves were expected to increase their latency to touch locations with increased distance from the rewarded cue. We used a linear mixed model to explore the fixed effect of location on response latency, with day specified as a fixed effect and calf specified as a random effect.

We used latency to touch ambiguous locations to calculate the pessimism score. Latencies to touch ambiguous locations were corrected for activity by subtracting the time taken to reach each ambiguous location from the time taken to reach the rewarded one. The pessimism score was obtained by averaging the time taken to touch each location on the two days of testing. We did not have any *a priori* predictions on whether any specific location would be of particular interest. Therefore, we averaged response to all three ambiguous locations to provide a reliable estimate of how calves respond to ambiguity overall. We considered calves to be more pessimistic when they displayed greater overall latencies to touch the ambiguous cues, similarly to previous studies on calves [29] and other species [33].

Of the 20 calves enrolled, two animals did not drink the sweet solution and one animal was an extreme outlier (increasing sweet solution consumption by 225% to 1600% compared to baseline values on the days following disbudding; Dixon test Q = 0.65, *P* < 0.001); these animals were removed from the analyses, leaving a total of 17 calves.

Baseline consumption of the sweet solution was calculated by averaging intakes from day 42 to 44 (i.e. the 3 last days before disbudding; Fig 1).

We first explored whether body weight affected baseline consumption of the sweet solution using a linear regression. Then, to assess whether pain associated with disbudding would reduce the consumption of the sweet solution (i.e. anhedonia), we compared intakes before (baseline) and after disbudding (day 45) using a linear mixed model including group pen as an additional fixed factor. Data were log-transformed to normalize differences.

To explore whether pessimism affected the change in sweet solution intake on the day of disbudding, we first calculated the percentage change relative to individual baseline consumption and explored whether this was explained by variation in Pessimism using linear regression. Log transformation improved the distribution of residuals and was thus applied. We expected that some calves would return to their baseline intake in the days following the procedure and that pessimism would affect this recovery. To test this idea, we ran a linear mixed model using the sweet solution consumption as response variable, and baseline consumption, day (45 to 49), group pen, and pessimism as fixed effects, and calf as random effect. To normalize the distribution of residuals, sweet solution consumption and baseline consumption were log-transformed.

## Results

### Response to judgment bias tests

**Fig 2:**
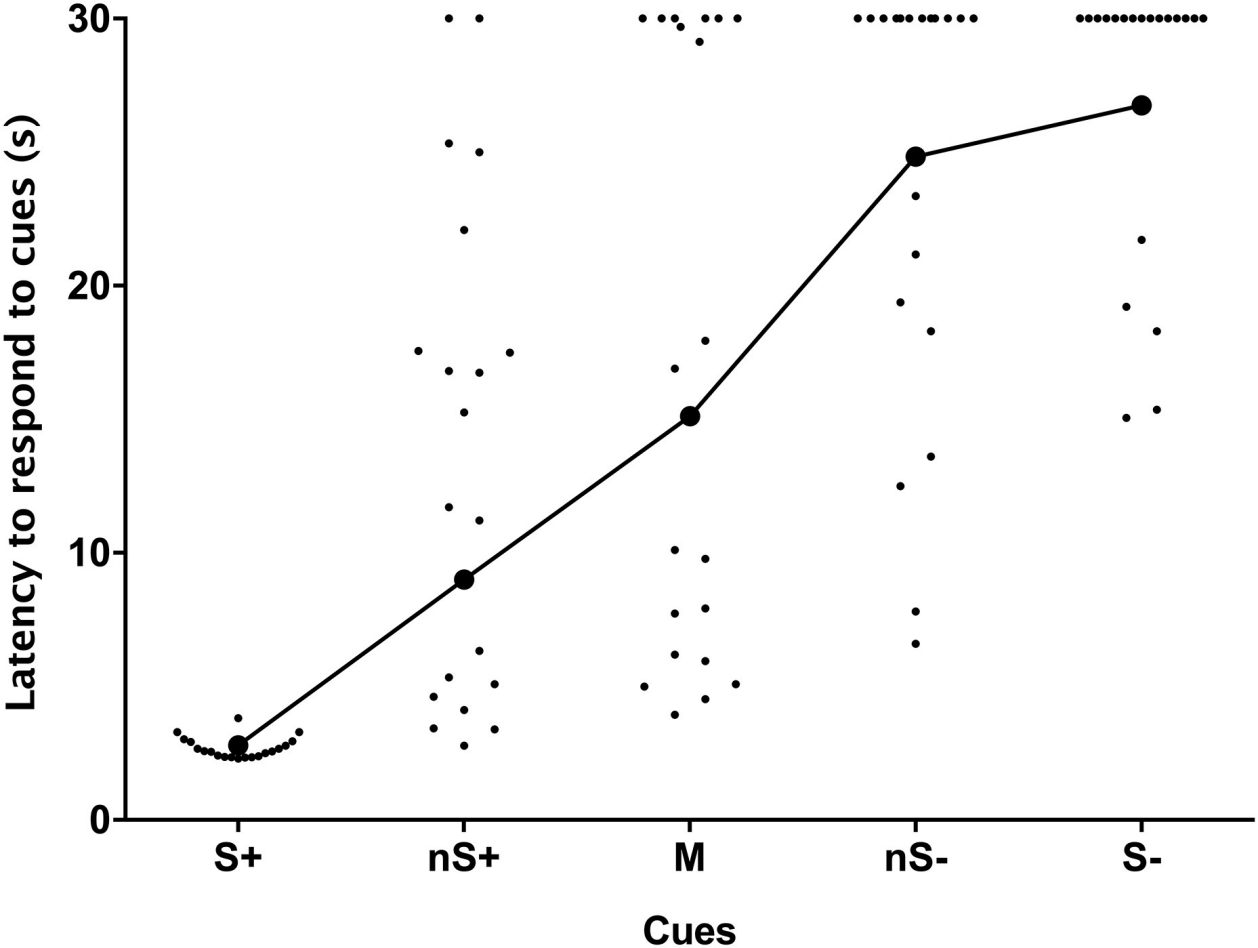
Latencies (raw data; mean ± SE) to touch the different locations of the judgment bias tests. Calves (n = 20) were trained to associate one side with a reward and the other side with a mild punishment. Once trained, calves were tested by presenting them with three ambiguous locations (nS+, M and nS-) between the two conditioned locations. Each point represents the averaged measure collected over the two days of testing for each calf.

Location had a strong effect on latency to touch the bottles (F_4,76_ = 45.12, *P* < 0.0001, Fig 2), indicating that calves successfully generalized their response from the reinforced locations, with no effect of test day (*P* > 0.05).

### Pain-induced anhedonia

**Fig 3:**
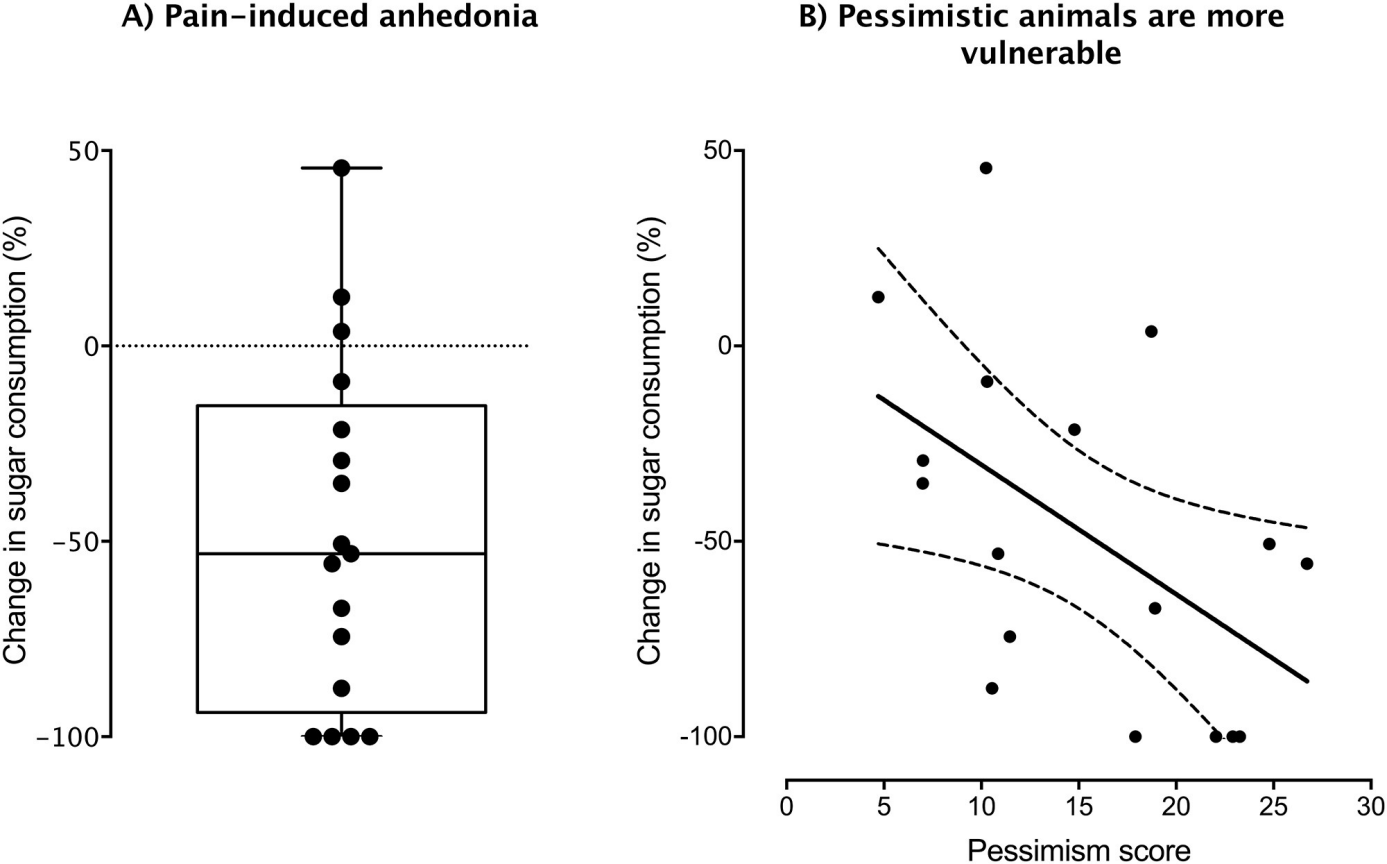
Panel A) shows the change (%) in sweet solution intake in calves (n = 17) on the day of hot-iron disbudding relative to their baseline intake (calculated as the average of the 3 d preceding disbudding). Panel B) shows the relationship between pessimism score and the change in sweet solution intake on the day of disbudding (dashed curves represent the Cl_95%_ bands). Raw data are presented.

Calves consumed (mean ± SD) 2.01 ± 2.19 kg/d of sweet solution before disbudding. Body weight did not relate to consumption of the sweet solution before disbudding (*P* > 0.05). All but 3 of the 17 calves reduced their sweet solution intake on the day of disbudding. Intake was reduced on average by 48.4 ± 44.3% (F_1,16_ = 18.17, *P* < 0.001; Fig 3A), and more pessimistic animals showed greater declines in intake of the sweet solution on the day of disbudding (*R*^2^ = 0.28, *P* = 0.029; Fig 3B).

**Fig 4:**
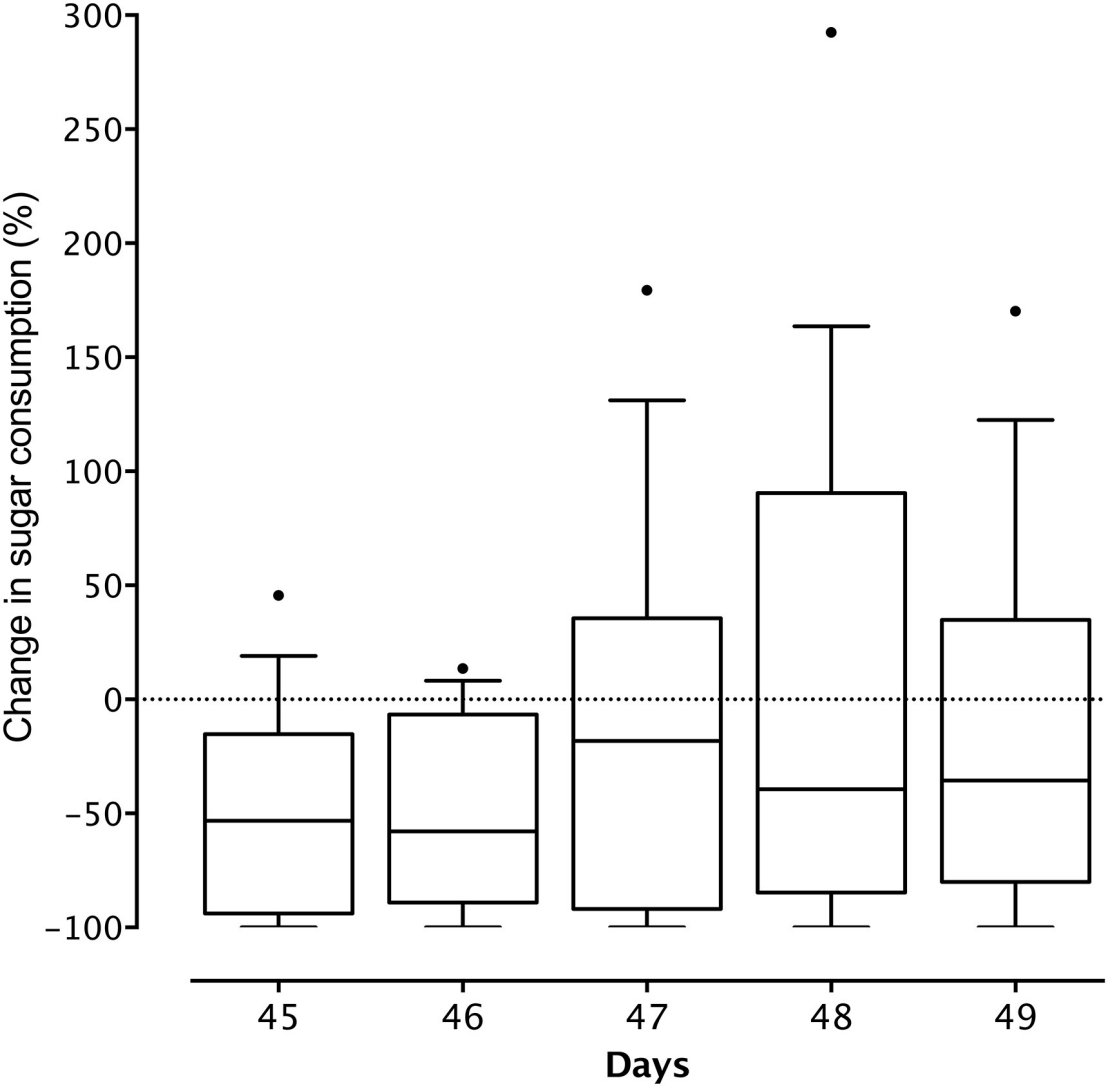
Changes (%) in sweet solution intake over the five days following hot-iron disbudding in calves (n = 17). Changes were calculated relative to their baseline intake (average of the 3 d preceding disbudding). Calves were disbudded on day 45. Boxes indicate the interquartile ranges with the median, whiskers indicate the 10^th^ and 90^th^ percentiles and outliers are represented by dots. Raw data are presented.

Baseline sweet solution intakes strongly affected intakes following disbudding (F_1,14_ = 43.45, *ß* = 0.54, *P* < 0.001) but no changes were detected over the 5 days (*P* > 0.05), indicating that calves did not recover from the initial drop in sweet solution intake over this period (Fig 4). More pessimistic animals tended to drink less (F_1,15_ = 3.91, *ß* = − 0.007, *P* = 0.07) during the post-operative period.

## Discussion

Calves showed evidence of reduced consumption of a sweet solution after disbudding, indicating that the procedure may have induced anhedonia for days. More pessimistic animals showed more evidence of pain-induced anhedonia, suggesting that these animals were more affected.

Given the lack of self-reports in non-human animals (and in some humans), the affective consequences of pain must be explored using other methodologies. Anhedonia may be especially useful for making inferences about the affective component of pain and other negative affective states [34]. Earlier studies have reported pain-induced anhedonia in rats (inflammatory pain: [35], chronic pain: [36]), and in humans [37, 38]. A recent study from our group found that calves were slower to access a milk reward 6 h after disbudding when tested in a judgment bias test, suggesting a motivational deficit consistent with anhedonia [17]. The current study specifically examined changes in consumption of a sweet solution to confirm this hypothesis. Taken together, the results of both studies are consistent with calves attributing lower value to a reward after hot-iron disbudding (i.e. anhedonia), although anorexia cannot be ruled out as calves may have considered the sweet solution as part of their diet. Little is known regarding the hedonic value associated with the consumption of milk compared to other sweet solutions in calves. Motivation for resources other than sweet solutions (e.g. non-food rewards) may allow stronger inferences. For instance, we recently found that weaned calves reduce their use of a mechanical brush after social mixing [39].

Due to technical issues, we were not able to collect milk and concentrate intakes for this study. Measuring these intakes would have allowed us to compare intake of the sweet solution with that from other feed sources, and thus help disentangle anhedonia from anorexia. One previous study showed a reduction in motivation to access a milk reward [17], but other work exploring variation in milk intakes after disbudding did not find differences between disbudded and sham calves, even when the procedure was carried out without pain control [40]. Taken together, these results suggest that hot-iron disbudding does not induce anorexia.

How disbudding affects social behavior is poorly understood. Given that the sweet solution was offered using only one teat per pen, social facilitation and competition may have affected our results. No effect of pen was noted in the statistical analyses, and no obvious signs of competition were observed, but future studies should account for this or allow multiple calves to drink at once. It is possible that pain changes calf motivation for social contact. For instance, a recent study in mice showed that chronic pain increased social avoidance after repeated social defeat [41]. If pain increased social avoidance in calves this may have led to a reduction in feeding at busy times.

The current study used a within-subject design where each calf was its own control. We expected that calves would decrease their consumption after hot-iron disbudding before returning to baseline levels in the subsequent days. As expected, calves reduced their consumption after disbudding, but on the days following the procedure some calves appeared to recover while others did not. The calves that failed to return to baseline consumption may have experienced more persistent pain. Future studies should explore calves’ consumption over a longer period of time.

Whether hot-iron disbudding induces long-lasting pain has received little attention [42]. Most studies using wound-directed behaviors and cortisol plasma levels did not explore evidence of pain beyond 24 h [14, 42]. Some calves experienced reduced consumption of the sweet solution for as long as 5 days, a result consistent with other recent reports showing long-lasting pain after hot-iron disbudding [43–45]. These results, along with the negative judgment bias observed after disbudding in previous studies [16, 46], suggest that the procedure may induce persistent pain potentially leading to depressive-like mood in dairy calves. These results indicate that calves should be provided effective post-operative medication (e.g. NSAIDs) to mitigate the aversiveness of the procedure [47] and restore appetite [48].

The current study does not allow strong inferences specific to pain; it is possible that other affective states associated with the procedure may also have contributed to the calves’ responses. To allow for stronger inferences specific to pain, future research could consider the addition of a sham group to better control for the non-pain related aspects of the procedure. This would have also allowed to control for any effects of age and stress associated with the procedure (e.g. sedation) on sweet solution consumption. We do not think that these factors could account for the almost 50% decline in intake on the day following disbudding, especially given that the rewarding properties of sucrose are known to increase with age in calves [18]. The reduced consumption following disbudding might also have been associated with the drug used to sedate the calves. However, the behavioral and physiological effects of xylazine are known to wane after 1 h [49], so we consider it unlikely that this affected response to sucrose 6 h after the procedure (i.e. when calves were allowed access to the sweet solution), especially given that the reduced intakes persisted on the following days.

Little work has explored individual differences in pain responses originating from psychological or cognitive processes in animals. In the current study, calves varied in their responses (i.e. changes in sweet solution intake) following hot-iron disbudding. The greater decline in sweet solution in pessimistic animals suggests that they were most affected by the procedure. This result is consistent with studies in humans in which pessimistic people reported worse expectations about future pain [50], more pain in a cold pressor task [9] and after surgery [51]. Furthermore, artificially induced optimism lowered pain intensity ratings suggesting a causal relationship [52].

Pessimism may negatively interact with pain-specific expectations that are known to affect the pain response [53] or may have deleterious effects on how people cope with pain, notably by increasing catastrophizing. Studies to date have not found an interaction between general expectations and pain-specific expectations [9, 52] but found an effect on pain catastrophizing [9, 54]. Calves may have had different pain experiences before our study (e.g. at birth, painful gastro-intestinal diseases), and these experiences may have affected their responses to future pain. Alternatively, pessimistic calves might be more vulnerable to pain because of poorer coping abilities. This interpretation is consistent with previous studies showing that pessimistic animals were more vulnerable to stressors [27, 31]. For instance, stress-induced anhedonia was stronger and lasted longer in pessimistic rats [27] that were also found more sensitive to negative feedback [55]. The negative expectations of pessimistic individuals are likely to contribute to the experience of negative feelings after painful or stressful experiences and to play a role in the development and maintenance of depressive symptoms such as anhedonia [30].

## Conclusion

Calves display signs of anhedonia for days after hot-iron disbudding, and this response is most pronounced in pessimistic animals. Prolonged anhedonic states are consistent with the long-lasting affective effects of pain and stress associated with this procedure, and highlights the vulnerability of more pessimistic animals. Hot-iron disbudding may thus have persistent negative consequences on the welfare of dairy calves.

## Supporting information

S1 Video Calf response during a test trial showing an ‘optimistic’ response. The calf has been trained to associate a reward on the right side and a punishment on the left. In this case, the calf approaches a bottle positioned at a test location intermediate (near-negative location) to the two reinforced locations on either side of the apparatus.
(M4V)

S2 Video Calf response during a test trial showing an ‘pessimistic’ response. The calf has been trained to associate a reward on the right side and a punishment on the left. In this case the calf avoids a bottle positioned at a test location intermediate (near-negative location) to the two reinforced locations on either side of the apparatus.
(M4V)

S1 File Stats codes. (DOCX)

S1 Dataset (XLSX)
