# Supplementary material for: Pessimistic dairy calves are more vulnerable to pain-induced anhedonia
Source: PLoS One. 2020 Nov 18;15(11):e0242100. doi: 10.1371/journal.pone.0242100 (PMC7673544; doi:10.1371/journal.pone.0242100)
Supplement: S1 File — (DOCX) [file pone.0242100.s003.docx]

**S1 File. Stats codes**

**Pessimistic dairy calves are more vulnerable to pain-induced anhedonia**

Benjamin Lecorps, Emeline Nogues, Marina A.G. von Keyserlingk, Daniel M. Weary ^*^

Animal Welfare Program, Faculty of Land and Food Systems, 2357 Main Mall, University of British Columbia, Vancouver BC V6T 1Z6

*Corresponding author: [danweary@mail.ubc.ca](mailto:danweary@mail.ubc.ca)

**Codes used in R for outlier detection**

***# Package to install***

library(outliers) # load the package

***# Data loading***

Pain_induced_anhedonia<-read.csv(file.choose(), sep=";", dec=",") # File opening/loading

Sugar_day45<-subset(Pain_induced_anhedonia,Day=="45") # Subset containing disbudding day only

**# *Outlier test***

dixon.test(Change, data = Sugar_day45, opposite = FALSE, two.sided = TRUE)

**# *Outcome of the test***

#Q = 0.65453, p-value < 2.2e-16

#alternative hypothesis: highest value 225.6410256 is an outlier

**Codes used in SAS**

FILENAME REFFILE 'C:\.../Pessimism.xlsx';

**PROC** **IMPORT** DATAFILE=REFFILE

DBMS=XLSX

OUT=Pessimism;

GETNAMES=YES;

**run**;

**data** numbers;

set Pessimism;

**run**;

**proc** **univariate** plot normal;

var latency;

**run**;

**run**;

**PROC** **CONTENTS**;

**RUN**;

* Validation of the judgment bias test (effects of distance from rewarded cue);

**proc** **mixed** data=Pessimism;

class Calf Distance Day;

model latency= Day Distance Day*Distance/residual;

repeated /sub=Calf type=ar(**1**);

lsmeans Day Distance Day*Distance / diff cl ;

**run**;

FILENAME REFFILE 'C:\.../Pessimism_anhedonia.xlsx';

**PROC** **IMPORT** DATAFILE=REFFILE

DBMS=XLSX

OUT= Pessimism_anhedonia;

GETNAMES=YES;

**run**;

**data** numbers;

set Pessimism_anhedonia;

**run**;

**PROC** **CONTENTS**;

**run**;

**proc** **sort**;

by Day;

**run**;

**proc** **univariate** ;

var Sugar;

**run**;

*Removal of the outlier and animals that did not drink;

**data** Pessimism_anhedonia;

set Pessimism_anhedonia;

if Calf in ('8027', '8030','8036') then delete;

**run**;

*Creating the different subsets;

*Baseline consumption (average of day 42, 43 and 44) is coded as Day 100;

**data** Baseline;

set Pessimism_anhedonia;

if Day in ('40','41','42','43','44','45','46','47','48','49') then delete;

**run**;

**data** Baseline_disbudding_day;

set Pessimism_anhedonia;

if Day in ('40','41','42','43','44','46','47','48','49') then delete;

**run**;

**data** Disbudding_day;

set Pessimism_anhedonia;

if Day in ('40','41','42','43','44','46','47','48','49','100') then delete;

**run**;

**data** post_disbudding;

set Pessimism_anhedonia;

if Day in ('40','41','42','43','44','100') then delete;

**run**;

*Relationship between sugar consumption and body weight before disbudding (see additional results);

**proc** **reg** data= Baseline;

model Log_Sugar = Body_weight ;

**run**;

*Effect of disbudding on sugar consumption;

**Proc** **mixed** data = Baseline_disbudding_day;

Class Calf Group;

Model Log_Sugar = Day Group / solution residual htype=**1** ;

Repeated / subject =Calf type=ar(**1**);

Lsmeans ;

**run**;

*Relationship between Pessimism score and the change in sugar consumption on disbudding day (day 45);

**proc** **reg** data= Disbudding_day;

model Pessimism = Change ;

**run**;

*Effect of day, baseline consumption and Pessimism on post-disbudding days (days 45 to 49);

**Proc** **mixed** data = Post_disbudding ;

Class Calf Group ;

Model Log_Sugar = baseline_log_sugar day Pessimism Group / solution residual htype=**1** ;

Repeated / subject =Calf type=ar(**1**);

Lsmeans ;

**run**;
